# When curiosity counts more: team curiosity, transactive memory systems, and team formalization

**DOI:** 10.3389/fpsyg.2026.1796809

**Published:** 2026-04-24

**Authors:** Shengjun Zhang, Junying Cao, Yige Zhuo

**Affiliations:** 1 Department of Human Resource Management, Shanghai University of Finance and Economics, Shanghai, China; 2 Business School, Nanjing University, Nanjing, China; 3 Business School, Jiangsu Open University, Nanjing, China; 4 School of Public Economics and Investment, Shanghai University of Finance and Economics, Shanghai, China

**Keywords:** curiosity, team, team formalization, team performance, transactive memory system (TMS)

## Abstract

While curiosity has been widely studied at the individual level, its relevance to team functioning remains largely underexplored. Drawing on transactive memory system (TMS) theory, we propose that team curiosity—defined as the average level of curiosity among team members—enhances team performance by fostering the development of TMS within teams and that this indirect effect is moderated by team formalization (the extent to which team structures, roles, and routines are explicitly codified). We test our hypotheses using multi-source, time-lagged data from 87 teams across multiple organizations. The results indicate that team curiosity is positively related to the presence of TMS, which, in turn, predicts team performance. Importantly, the indirect effect of team curiosity on team performance via TMS is stronger among teams with higher levels of formalization. These findings contribute to the literature on curiosity by introducing team curiosity as a novel team-level construct, identifying its effect on team performance through TMS, and revealing how structural conditions shape its effectiveness.

## 1 Introduction

In today's dynamic and knowledge-intensive work environments, teams are increasingly expected to explore novel ideas, integrate distributed expertise, and adapt to evolving challenges (Harvey et al., 2022). To meet these expectations, curiosity stands out as a fundamental psychological force that drives exploration, information seeking, and adaptation (Lievens et al., 2022). Despite growing interest in curiosity as an organizational behavior, research has predominantly focused on its effects at the individual level, specifically its impact on individuals' behavioral intentions, work performance, creativity, and interpersonal relationships (Fath et al., 2022; Hagtvedt et al., 2019; Harrison and Dossinger, 2017; Mussel, 2013; Polman et al., 2022; Schweitzer et al., 2023; Thompson et al., 2023). As a result, we know surprisingly little about how curiosity operates at the team level and how it influences teams' emergent states and outcomes.

In this study, we address this gap in the literature by theorizing and empirically examining the construct of team curiosity and its implications for team performance. We define team curiosity as the average level of curiosity across team members, which captures the extent to which a team—as a collective—is composed of individuals who are prone to engage in exploration and information seeking. Drawing from research on transactive memory systems (TMS) (e.g., Lewis, 2003; Ren and Argote, 2011), we propose that team curiosity is associated with higher team performance through the development of shared cognitive structures that enable effective knowledge specialization, credibility, and coordination among team members (i.e., TMS). In doing so, we shift attention from the individual-level conceptualization of curiosity to a team-level property that supports team function and performance.

We further propose that the relationship between team curiosity and TMS is contingent on team formalization—the extent to which formal rules, standardized policies, and explicit procedures govern team decision-making processes and working relationships (Fredrickson, 1986). While formalization can restrict spontaneous knowledge sharing (Amber et al., 2019; Schepers et al., 2019), we argue that it also provides a structure within which curiosity-driven behaviors may become particularly instrumental. More specifically, with a high degree of formalization, curious team members may work harder to overcome structural rigidity, making curiosity more consequential for the emergence of TMS in teams. This leads us to propose a moderated mediation model in which team curiosity indirectly enhances performance via TMS, with this indirect effect being stronger under conditions of high formalization.

**Figure 1:**
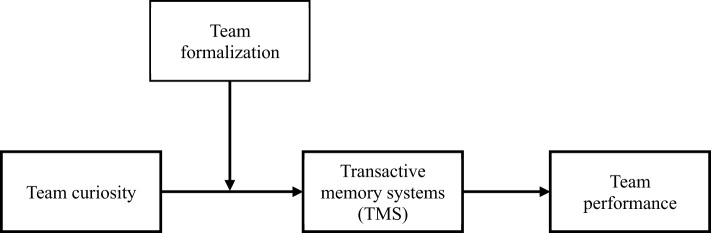
Theoretical model.

Our research (summarized in Figure 1) makes several contributions. First, we extend the broader literature on curiosity by introducing and validating team curiosity as a meaningful collective construct that predicts a team's emergent cognitive state and performance. Thus, this study responds to the call for exploring the effects of curiosity at the collective level (Kashdan et al., 2023; Lievens et al., 2022). Second, we contribute to the TMS literature by identifying team curiosity as a novel antecedent that motivates the formation of shared knowledge systems. Finally, we offer new insights into team formalization by demonstrating that curiosity may serve as a compensatory mechanism in structurally rigid teams.

## 2 Theory and hypotheses

### 2.1 Team curiosity

In modern organizational settings, teams are frequently expected to engage in adaptive learning, exploratory searches, and novel problem solving (Harvey et al., 2022). These behaviors are fundamentally underpinned by curiosity—a motivational tendency to seek new knowledge, embrace uncertainty, and explore unfamiliar ideas (Litman, 2008). Although curiosity has traditionally been conceptualized at the individual level, understanding how teams engage in collective exploration requires extending this construct to the team level (Lievens et al., 2022).

We conceptualize team curiosity as the average level of curiosity among team members, capturing the extent to which a team is collectively composed of individuals who are inclined to seek new knowledge, question assumptions, and engage in exploratory thinking. This operationalization follows a compositional aggregation logic (Chan, 1998), whereby individual-level attributes are meaningfully combined to represent higher-level constructs when they reflect a pooled resource available to the collective. In this sense, team curiosity represents a distributed epistemic motivational resource that resides in the team by virtue of its members.

We focus on the mean level of curiosity because our theoretical interest lies in the team's overall capacity to initiate and sustain inquiry-driven behaviors that support collective knowledge development. When a larger proportion of team members possess higher levels of curiosity, the likelihood of question asking, information seeking, and knowledge probing increases, thereby facilitating exploratory interactions within the team. Thus, the average level of curiosity captures the extent to which such epistemic engagement is broadly supported and enacted across team members.

Importantly, this conceptualization does not require team members to exhibit uniform levels of curiosity. Team curiosity is not conceptualized as a shared perception or emergent state, but rather as the aggregate availability of curiosity-driven tendencies within the team. Even when curiosity is unevenly distributed—for example, when a team includes one highly curious member alongside less curious others—a higher average level still indicates a greater overall supply of epistemic motivation that can be mobilized in team interactions. Prior research has similarly demonstrated that team-level means of individual traits, such as openness to experience (Homan et al., 2008; Bradley et al., 2013), positive affectivity (Kaplan et al., 2013), and emotional intelligence (Zhang et al., 2020), can meaningfully shape team processes and outcomes, even in the presence of substantial within-team variability.

At the same time, we acknowledge that teams may differ not only in their average level of curiosity but also in how curiosity is distributed among members. Such variability may influence interaction dynamics in distinct ways. However, our theoretical focus is on the collective epistemic capacity of the team, rather than the configuration of that capacity across individuals. Accordingly, we treat within-team variability in curiosity as a control variable, allowing us to isolate the unique effect of average team curiosity beyond differences in dispersion. Future research may build on this distinction by examining how different configurations of curiosity within teams shape team processes and outcomes.

Taken together, this conceptualization provides a theoretically grounded basis for examining how team curiosity, as a compositional resource, influences team-level cognitive structures such as transactive memory systems.

### 2.2 Team curiosity and the development of transactive memory systems

TMS represents shared cognitive structures within teams that enable members to specialize in distinct knowledge domains, develop credibility in one another's expertise, and coordinate effectively to access and integrate distributed knowledge among members (Bachrach et al., 2019). A foundational premise of a TMS is the recognition of “who knows what” within the team, which relies on active inquiry, information sharing, and cognitive interdependence (Ren and Argote, 2011). We argue that team curiosity, ora team's average tendency to seek novelty, question assumptions, and explore new ideas, is likely to play a critical role in fostering such behaviors, thereby contributing to the emergence and strengthening of a TMS.

Importantly, we do not assume that curiosity directly translates into the existence of a TMS. Rather, curiosity operates through specific epistemic behaviors that serve as micro-foundations for TMS development. Curiosity motivates individuals to ask questions, seek explanations, and probe the knowledge held by others (Litman, 2008). Through these inquiry-driven interactions, team members gradually uncover “who knows what,” evaluate the credibility of others' expertise, and learn how to coordinate access to distributed knowledge (Lievens et al., 2022). In this sense, curiosity is likely to contribute to TMS not by itself, but by stimulating the interaction patterns necessary for mapping, validating, and integrating team members' expertise. These processes are essential for the development of the three core dimensions of TMS, that is, specialization, credibility, and coordination (Bachrach et al., 2019; Ren and Argote, 2011).

While other constructs such as learning orientation and communication norms may also facilitate knowledge exchange, curiosity is conceptually distinct in its role as an epistemic driver of inquiry (Lievens et al., 2022). Learning orientation reflects a general desire to develop competence (Vandewalle et al., 2019), whereas communication norms reflect shared expectations regarding how interaction is structured and enacted within a team (Yates and Orlikowski, 2002). In contrast, curiosity directly motivates individuals to seek out unknown information, question assumptions, and actively probe others' expertise (Lievens et al., 2022). As such, curiosity is uniquely positioned to stimulate the discovery and mapping of distributed knowledge, which lies at the core of TMS development, rather than merely facilitating general information exchange.

In addition to these inquiry-driven mechanisms, team curiosity may also indirectly support TMS development by fostering a psychologically safe environment that encourages intellectual risk-taking and information exchange (Edmondson and Bransby, 2023). Such environments reduce the social cost of disclosing ignorance and seeking help, thereby facilitating stronger knowledge coordination and more effective functioning of a TMS (Hood et al., 2016).

In summary, by catalyzing exploratory interaction patterns, increasing epistemic openness, and promoting distributed knowledge awareness, team curiosity is likely to facilitate the development of the cognitive and relational infrastructure necessary for the development of TMS in teams.

**H1**. Team curiosity is positively related to the development of TMS in teams.

### 2.3 Transactive memory systems and team performance

As teams increasingly serve as the fundamental units for executing complex and interdependent organizational tasks, their ability to effectively manage and integrate distributed knowledge is critical for performance. A TMS facilitates such management and integration by providing a collective cognitive infrastructure in which individual expertise is differentiated (specialization), trusted (credibility), and coordinated (coordination) across the team (Lewis, 2003; Wegner, 1987). This infrastructure enables teams to efficiently store, retrieve, and apply knowledge distributed among its members, enhancing both the quality of decisions and the execution of tasks.

A TMS improves team performance through several interrelated mechanisms. First, specialization enables team members to focus on their areas of expertise without duplicating knowledge, thereby reducing cognitive redundancy and maximizing the utility of the team's knowledge base (Ren and Argote, 2011). Second, credibility enhances the speed and quality of information exchange among team members, as they are more likely to rely on information provided by those perceived as experts, reducing the need for excessive verification or second-guessing (Lewis, 2003). Third, coordination streamlines collaborative processes by clarifying roles and expectations, thus facilitating the integration of diverse perspectives (Bachrach et al., 2019).

Empirical studies have consistently found that TMS is positively associated with task performance. For example, through a survey of 104 work teams, Zhang et al. (2007) found that TMS had a positive effect on team performance. Based on 132 four-person teams, Lee et al. (2014) found that TMS was positively related to team performance. A recent meta-analysis also highlighted the positive relationship between TMS and team performance (Bachrach et al., 2019).

By combining Hypothesis 1 with the premise linking TMS to team performance, we argue that a higher average level of team curiosity increases the likelihood of a team developing a TMS and, in turn, achieving stronger performance. Thus, we propose the following hypothesis:

**H2**. Team curiosity has a positive indirect effect on team performance via TMS.

### 2.4 The moderating role of team formalization

While team curiosity provides the motivational foundation necessary for exploratory behavior and cognitive integration within teams, the extent to which curiosity fosters the development of a TMS critically depends on the structural context in which the team operates. Team formalization, defined as the extent to which formal rules, standardized policies, and explicit procedures govern team decision-making processes and working relationships (Fredrickson, 1986), is a crucial structural feature that influences this relationship. Prior research has yet to recognize formalization as a dual-edged structural characteristic that, despite reducing ambiguity and clarifying role expectations, may simultaneously constrain spontaneous communication, informal knowledge exchange, and interpersonal interactions (Amber et al., 2019; Schepers et al., 2019). Formalized team contexts often impose rigid communication protocols and strict operational guidelines, potentially suppressing the casual interactions and organic exchanges necessary for team members to effectively discover and leverage one another's specialized knowledge and expertise (Mao et al., 2009).

However, we argue that in such structurally constrained environments, team curiosity is particularly consequential as a critical compensatory mechanism. By its nature, curiosity involves proactive knowledge-seeking behaviors, a willingness to engage in inquiry, and openness to new information, all of which are especially beneficial when formal structures limit spontaneous dialogue and flexibility. More specifically, curious team members actively initiate discussions, question established norms, and engage in exploratory conversations that help the team overcome formalization-imposed rigidity (Lievens et al., 2022). By explicitly probing teammates' areas of expertise and engaging in knowledge exchange, curious teams are more likely to navigate and penetrate the structural boundaries imposed by formal rules. Consequently, these curiosity-driven behaviors can lead to a generally higher level of team awareness of “who knows what,” thereby directly supporting the development of TMS dimensions like specialization, credibility, and coordination (Ren and Argote, 2011).

Moreover, we posit that team formalization may actually amplify the functional value of curiosity-driven behaviors. Amid a high level of formalization, curiosity-oriented actions by team members stand out and gain strategic importance precisely because alternative pathways for spontaneous information exchange are generally limited or blocked (Mao et al., 2009). Such structural rigidity increases the salience and necessity of deliberate, curiosity-based interactions aimed at clarifying roles, uncovering hidden expertise, and facilitating the cognitive coordination of specialized knowledge (Lievens et al., 2022). Thus, while formalization in isolation often negatively impacts the formation of shared cognitive structures, high team curiosity effectively counterbalances or mitigates these adverse effects by fostering focused, intentional, and inquiry-based communication.

In summary, although team formalization may negatively affect TMS development in general, we propose that high levels of team curiosity can buffer or even reverse this effect. Formalization can serve as a boundary condition that amplifies the importance and impact of curiosity-driven behaviors in enabling the emergence of a TMS in teams.

**H3**. Team formalization moderates the relationship between team curiosity and TMS, such that the positive relationship is stronger when team formalization is high.

We have proposed that team curiosity interacts with team formalization such that the positive relationship between team curiosity and TMS is stronger at high levels of team formalization. Moreover, we have posited that team curiosity exerts a positive indirect effect on team performance via TMS. Building on these propositions, we now advance an integrated moderated mediation model in which TMS serves as the mediating link between team curiosity and team performance, with the strength of this indirect effect depending on the level of team formalization. This rationale rests on the idea that the performance benefits of team curiosity are realized and transmitted through the development of TMS, particularly when formalized structures provide the conditions under which curiosity-driven behaviors are most effectively leveraged. Accordingly, we propose the following hypothesis:

**H4**. The indirect effect of team curiosity on team performance via TMS is stronger when team formalization is high.

## 3 Method

### 3.1 Sample and procedure

We sampled work teams comprising full-time employees from 12 firms in China across a wide range of industries (e.g., construction, electronics and technology, publishing, plastics, flooring) and job functions (e.g., marketing, accounting, RandD) to enhance the external validity of the proposed relationships. To ensure an adequate survey response rate, we first engaged in preliminary communication with senior management at each firm. Upon obtaining their approval, we coordinated with human resources managers to understand each company's organizational structure, finalize the timing and method of survey administration, and identify eligible participants. Trained research assistants explained the purpose of the study to each participant, emphasizing that all responses would remain confidential and used exclusively for academic research. To encourage participation, respondents received a small monetary incentive—approximately one U.S. dollar—for each completed survey round.

To mitigate common method bias, data were collected from team members and their leaders at three points in time. At Time 1 (T1), 464 team members and 97 team leaders (out of the 469 members and 103 leaders of 103 teams initially invited) completed the survey. At this stage, team members reported their levels of curiosity and demographic information, while leaders provided assessments of task interdependence and task formalization. 2 weeks later, at Time 2 (T2), 438 members from the same 103 teams reported their perceptions of their team's TMS. At Time 3 (T3), 4 weeks after T1, 87 team leaders provided team performance ratings. Each participant was assigned a unique five-digit identifier, and each team was assigned a unique three-digit code to facilitate data matching across the three time points. After matching the responses across all three waves, the final sample consisted of 87 team leaders (response rate = 86.14%) and 406 team members (response rate = 86.57%) across 87 teams. Among team members, 64.76% were between 26 and 40 years old, 48.39% were male, 82.63% held at least a college degree, and 73.45% had worked as part of their current team for at least 1 year. Team sizes ranged from 2 to 15 members, with a mean of 4.71.

### 3.2 Measures

All surveys were administered in Chinese, and we used Brislin's (1980) translation–back translation procedure to ensure semantic equivalence between the original and Chinese versions. Specifically, measures were developed in English and translated into Chinese by two researchers fluent in both Chinese and English. The survey was then translated back into English and compared to the original version. Any inconsistencies were resolved by researchers fluent in both languages. Unless otherwise noted, the following scale was used for questions: 1 = strongly disagree; 6 = strongly agree.

#### 3.2.1 Team curiosity

At T1, team members reported their level of curiosity as an individual-level trait. Following Harrison and Dossinger (2017), we measured curiosity using Litman's (2008) five-item curiosity scale. Example items included “I enjoy exploring new ideas” and “I enjoy learning about subjects that are unfamiliar to me.” Cronbach's α was.86 for all five items. Consistent with our conceptualization of team curiosity as a compositional resource, we used the average of members' curiosity scores to represent the team-level construct. Because this construct reflects pooled epistemic motivational resources rather than a shared perception, high within-team agreement is not a theoretical prerequisite for aggregation (Chan, 1998). This approach aligns with past studies that also used average scores to aggregate team members' traits as a means of forming team-level measures (e.g., Bradley et al., 2013; Homan et al., 2008).

#### 3.2.3 Task formalization

At T1, team leaders provided ratings on their team's formalization using Hirst et al.'s (2011) three-item scale, which features items like the following: “There are a lot of rules and regulations in this team” (α = 0.72).

#### 3.2.4 Transactive memory system (TMS)

At T2, 2 weeks after T1, each team member was asked to rate their perceptions of TMS within their team using Lewis's (2003) fifteen-item scale. Some example items include “I know which team members have expertise in specific areas”, “I trusted that other members' knowledge about the project was credible” and “Our team had very few misunderstandings about what to do” (α = 0.93). We calculated the value of the within-group agreement index (r_wg_; James et al., 1993) and intraclass correlation coefficients (ICC1 and ICC2; Bliese, 2000) to justify the construct aggregation. The aggregation of individual members' responses to the team level was supported by the acceptable r_wg(j)_ mean value of.95, and median value of.97, with 97.70% of teams (*n* = 85) yielded a satisfactory level (0.70). The ICC1 was 0.12, and the ICC2 was 0.37, with a significant between-group effect (*F* = 1.60, *p* = 0.002). While the ICC2 value may initially seem relatively low, small average team sizes can naturally give way to low ICC2 values (Bliese, 1998). Multiple recent studies have suggested that ICC2 values greater than 0.25 are still acceptable in cases with high r_wg_ as well as significant F-test results (e.g., Chiu et al., 2022; Dietz et al., 2015). Accordingly, the aggregation of TMS values was justified.

#### 3.2.5 Team performance

At T3, 4 weeks after T1, each team leader rated their team's performance via Kirkman and Rosen's (1999) six-item scale, a sample item is “The work team is a productive team” (α = 0.93).

#### 3.2.6 Control variables

We included several control variables that could influence the development of TMS and team performance, including team size (Guimerà et al., 2005; Ren et al., 2006), team diversity (Bachrach et al., 2019; Bell et al., 2011) and team task interdependence (Somech et al., 2009; Zhang et al., 2007). More specifically, we controlled for team diversity generally by controlling for the factors of age, gender, educational background and team tenure. We categorized age into nine groups, ranging from 1 (25 years or younger) to 9 (over 60 years) with 5-year intervals in between. We measured gender as a binary variable (1 = male, 0 = female). We divided level of educational attainment into six categories: junior high school or below, technical secondary school or high school, junior college, bachelor's degree, master's degree and doctoral degree. We categorized team tenure into four groups, ranging from 1 (less than 1 year) to 4 (more than 3 years) with 1-year intervals in between. As these demographic characteristics were measured categorically, we calculated diversity using the Blau Index, defined as 1 minus the sum of the squared proportions of members in each category. Task interdependence was assessed at Time 1 by team leaders using Dean and Snell's (1991) five-item scale (α = 0.82). Moreover, considering our theoretical focus is on the team's collective epistemic capacity, rather than on the configuration of that capacity across members. Accordingly, we include within-team variability in curiosity—the standard deviation of the individual curiosity scores within each team—as a control variable in order to isolate the unique effect of average team curiosity beyond differences in dispersion.

### 3.3 Analytical strategy

We employed Mplus 8.3 to test our hypotheses. Given that teams were nested within specific firms, we applied the sandwich estimator to account for non-independence arising from multilevel clustering and to correct for potential estimation bias (Li et al., 2020; Muthén and Muthén, 2017). To examine the moderated mediation model, we followed the procedure outlined by Preacher et al. (2007). All control variables, the independent variable and the moderator were grand-mean centered. We constructed the interaction term by multiplying the centered values of team curiosity and team formalization (Aiken and West, 1991). As the indirect and conditional indirect effects involve compound coefficients that are not normally distributed, we conducted a Monte Carlo simulation in *R* with 20,000 iterations to generate bias-corrected confidence intervals (CIs) for the estimation of these effects (Edwards and Lambert, 2007; Preacher and Selig, 2012; Shrout and Bolger, 2002).

## 4 Results

Descriptive statistics and correlations are presented in Table 1.

**Table 1:** Descriptive statistics and correlations.

Variables		M	SD	1	2	3	4	5	6	7	8	9	10	11
1.	Team size	4.71	2.77	(—)										
2.	Age diversity (T1M)	0.56	0.16	0.29^**^	(—)									
3.	Gender diversity (T1M)	0.24	0.21	0.21^*^	0.11	(—)								
4.	Educational level diversity (T1M)	0.40	0.20	0.06	0.21	0.13	(—)							
5.	Team tenure diversity (T1M)	0.40	0.24	0.26^*^	−0.11	−0.02	−0.20	(—)						
6.	Team task interdependence (T1L)	3.94	0.93	0.03	−0.19	−0.06	−0.18	0.06	(0.82)					
7.	Curiosity standard deviation (T1M)	0.73	0.31	0.21^*^	0.09	−0.08	0.02	0.19	–.01	(—)				
8.	Team curiosity (T1M)	4.63	0.40	−0.20	0.12	0.15	−0.08	0.04	–.09	–.02	(0.86)			
9.	Team formalization (T1L)	3.70	0.97	−0.05	−0.11	0.08	−0.03	−0.02	0.19	0.00	0.11	(0.72)		
10.	TMS (T2M)	4.48	0.45	0.01	0.08	−0.01	0.12	0.05	–.05	–.09	0.37^***^	−0.18	(0.93)	
11.	Team performance (T3L)	4.95	0.84	0.14	0.22^*^	0.02	0.10	−0.04	–.02	−0.13	0.02	0.14	0.29^**^	(0.93)

*N* = 87. T1M = rated by team members at time1, T2M = rated by team members at time2, T1L = rated by team leaders at time1, T3L = rated by team leaders at time3; TMS = Transactive memory systems.

^*^p<*0*.05; ^**^p<*0*.01; ^***^p<*0*.001.

Prior to hypothesis testing, we sought to examine the study measure's discriminant validity. Two separate sets of confirmatory factor analyses (CFA) were conducted, as measures were provided by different sets of respondents (team members and team leaders), and common-method bias is most likely to occur when measures are rated by the same source. Supporting the distinctiveness of the measures (i.e., curiosity, TMS) rated by team members, the results showed that the two-factor model (i.e., the two employee-rated variables as two separate factors) provided a reasonable fit to the data (χ^2^ (19) = 61.42, CFI = 0.98, TLI = 0.97, RMSEA = 0.07, SRMR = 0.04) (Hair et al., 2019; Hu and Bentler, 1999) and a significantly better fit than the one-factor model (i.e., two variables combined as one factor) (Δχ^2^ (1) = 491.88, *p* < 0.001; CFI = 0.71, TLI = 0.59, RMSEA = 0.26, SRMR = 0.15). Upon testing whether the two variables (i.e., team formalization and team performance) evaluated by team leaders are distinct from each other, the results revealed that the two-factor model (i.e., team formalization and team performance as two separate factors) offered an acceptable fit to the data (χ^2^ (25) = 27.84, CFI = 0.99, TLI = 0.99, RMSEA = 0.04, SRMR = 0.04) and yielded a significantly better fit than the one-factor model (i.e., team formalization and team performance as a combined factor) (Δχ^2^ (1) = 55.15, *p* < 0.001; CFI = 0.88, TLI = 0.84, RMSEA = 0.16, SRMR = 0.11). Thus, the results provided support for the discriminant validity of measures collected from both team members and team leaders.

All hypotheses were tested in path-analytic models via Mplus 8.3. Hypothesis 1 posited that team curiosity would be positively related to TMS. As shown in Table 2, after including the controls, the effect of team curiosity on TMS was significant and positive (*b* = 0.42, *SE* = 0.13, *p* = 0.001), supporting Hypothesis 1. Hypothesis 2 posited that team curiosity would have a positive indirect effect on team performance via TMS. As shown in Table 2, after controlling for team curiosity and other related variables, we found TMS was positively related to team performance (*b* = 0.67, *SE* = 0.26, *p* = 0.009). Moreover, the Monte Carlo results revealed that the indirect effect of team curiosity on team performance via TMS was significant (estimate = 0.28, 95% CI = [0.05, 0.63]), lending further support to Hypothesis 2.

**Table 2:** Results of path analysis.

Variables	TMS (T2M)		Team performance(T3L)	
	*b*	*SE*	*b*	*SE*
Intercept	4.40^***^	0.11	1.80	1.23
Team size	0.02	0.01	0.03	0.02
Age diversity (T1M)	−0.14	0.18	1.08^*^	0.47
Gender diversity (T1M)	−0.16	0.24	−0.13	0.41
Educational level diversity (T1M)	0.41^*^	0.17	0.02	0.30
Team tenure diversity (T1M)	0.18	0.16	−0.08	0.46
Team task interdependence (T1L)	0.02	0.03	−0.02	0.10
Curiosity standard deviation (T1M)	−0.10	0.13	−0.36	0.30
Team curiosity (T1M)	0.42^**^	0.13	−0.32	0.27
Team formalization (T1L)	−0.15^**^	0.05	0.21^*^	0.09
Team curiosity^*^ team formalization	0.29^*^	0.12	0.06	0.24
TMS (T2M)			0.67^**^	0.26
R square	0.28^**^	0.09	0.22^**^	0.07

*N* = 87. T1M = rated by team members at time1, T2M = rated by team members at time2, T1L = rated by team leaders at time1, T3L = rated by team leaders at time3; TMS = Transactive memory systems. Unstandardized regression coefficients are reported.

^*^p<*0*.05; ^**^p<*0*.01; ^***^p<*0*.001.

**Figure 2:**
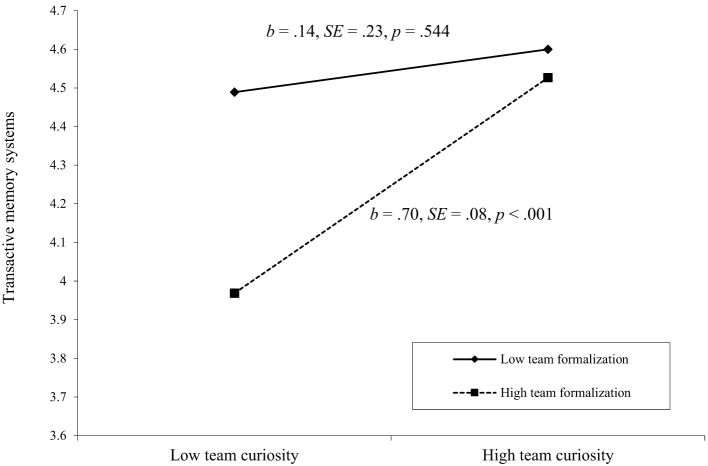
The moderating role of team formalization on the relationship between team curiosity and transactive memory systems.

Hypothesis 3 posited that team formalization would positively moderate the relationship between team curiosity and TMS, such that team formalization would amplify the positive effect of team curiosity on team performance. As shown in Table 2, the interaction between team curiosity and team formalization was significant and positively related to TMS (*b* = 0.29, *SE* = 0.12, *p* = 0.015). Simple slope analyses revealed that, amid a high level of team formalization (one standard deviation above the mean), the slope of team curiosity on TMS was positive and significant (*b* = 0.70, *SE* = 0.08, *p* < 0.001); amid a low level of team formalization (one standard deviation below the mean), the slope was not significant (*b* = 0.14, *SE* = 0.23, *p* = 0.544). The effect of the difference between these two conditions was significant (*b* = 0.56, *SE* = 0.23, *p* = 0.015). As shown in Figure 2, the relationship between team curiosity and TMS was more positive when team formalization was high, lending support to Hypothesis 3.

Hypothesis 4 proposed that the indirect effect of team curiosity on team performance via TMS would be moderated by team formalization. The Monte Carlo results revealed that the indirect effect of team curiosity on team performance via TMS was significant amid a high level of team formalization (estimate = 0.47, 95% CI = [0.10, 0.93]) but not significant amid a low level of team formalization (estimate = 0.09, 95% CI = [-0.22, 0.46]). The difference between these indirect effects was significant (estimate = 0.37, 95% CI = [0.03, 0.93]), lending support to Hypothesis 4.

For completeness, we also tested the direct effect of team curiosity on team performance. The results (see Table 3) indicated that the direct effect was non-significant (*b* = 0.09, *SE* = 0.24, *p* = 0.717, providing additional support for the indirect pathway through TMS. This finding is consistent with our theorized indirect effect model.

**Table 3:** Results of supplementary analysis.

Variables	Team performance(T3L)		Team performance(T3L)	
	*b*	*SE*	*b*	*SE*
Intercept	4.03^*^	1.69	4.74^***^	0.20
Team size	0.04	0.03	0.04	0.03
Age diversity (T1M)	0.94	0.45	0.99^*^	0.47
Gender diversity (T1M)	−0.23	0.52	−0.23	0.47
Educational level diversity (T1M)	0.31	0.38	0.30	0.37
Team tenure diversity (T1M)	−0.06	0.53	0.04	0.54
Team task interdependence (T1L)	0.03	0.11	−0.01	0.10
Curiosity standard deviation (T1M)	−0.48	0.36	−0.43	0.33
Team curiosity (T1M)	0.09	0.24	−0.04	0.26
Team formalization (T1L)			0.11	0.11
Team curiosity^*^ team formalization			0.25	0.23
R square	0.09	0.06	0.13^*^	0.06

*N* = 87. T1M = rated by team members at time1, T1L = rated by team leaders at time1, T3L = rated by team leaders at time3; Unstandardized regression coefficients are reported.

^*^p<*0*.05; ^**^p<*0*.01; ^***^p<*0*.001.

## 5 Discussion

In this study, we investigated TMS as a key explanatory mechanism underlying the positive association between team curiosity and team performance. Specifically, we examined the extent to which the indirect effect of team curiosity on team performance via TMS is contingent on the level of team formalization. Moreover, our analysis accounted for the effects of curiosity diversity within teams, enabling us to isolate the unique contributions of average team curiosity beyond intra-team variability. The results supported our theoretical model, demonstrating that teams with higher average levels of curiosity tend to develop stronger TMS and achieved higher levels of performance, particularly in teams with high levels of formalization.

Importantly, our supplementary analyses revealed that the direct effect of team curiosity on team performance was non-significant. This finding further reinforces our theorized indirect effect model, suggesting that team curiosity does not directly translate into performance outcomes. Instead, its influence appears to operate primarily through shaping the team's cognitive infrastructure, specifically, the development of transactive memory systems. In this sense, team curiosity functions as an enabling condition that facilitates the emergence of effective knowledge coordination processes, rather than as a direct driver of performance.

### 5.1 Theoretical implications

This study has made several important contributions to the literature on curiosity, TMS, and team formalization. First, we advanced the literature on workplace curiosity by introducing and theorizing curiosity as a team-level construct. Although curiosity has received growing attention in individual-level research—often being linked to behavioral intentions, work performance, creativity, and interpersonal relationships (Fath et al., 2022; Hagtvedt et al., 2019; Harrison and Dossinger, 2017; Mussel, 2013; Polman et al., 2022; Schweitzer et al., 2023; Thompson et al., 2023)—its collective manifestations and team-level consequences have largely gone unexamined. By aggregating individual curiosity to the team level, we conceptualized team curiosity not merely as a reflection of individual traits but as a compositional resource that shapes collective motivation and cognitive orientation. We demonstrated that team curiosity is associated with greater engagement in social-cognitive processes such as knowledge seeking and idea inquiry, which, in turn, strengthen team-level learning systems (i.e., TMS). In doing so, we expanded the literature's understanding of curiosity from a personal attribute to a distributed motivational property of teams, responding to the call for exploration of the effect of curiosity at the collective level (Kashdan et al., 2023; Lievens et al., 2022). Moreover, by identifying team formalization as a boundary condition, we contributed to a more nuanced account of team curiosity at its most consequential, particularly in settings where exploration is constrained by structural rigidity.

Second, we contributed to the TMS literature by identifying team curiosity as a previously unrecognized antecedent of TMS. While prior research has examined how task characteristics (e.g., Maynard et al., 2012; Zhang et al., 2007), team composition (e.g., Hood et al., 2016; Lazar et al., 2022; Pearsall and Ellis, 2006; Seong et al., 2015), leadership style (e.g., Bachrach et al., 2019), or team structure (e.g., Lee et al., 2014) influence TMS development, few studies have explored the motivational factors behind the formation of shared memory structures. Our study fills this gap in the literature by revealing that team curiosity—a collective tendency to seek, generate, and circulate novel information—can catalyze the behavioral patterns necessary to foster specialization, credibility, and coordination among team members. This insight will undoubtedly shift the TMS literature beyond structural or relational inputs, prompting it to incorporate motivational dynamics as key enablers of TMS emergence.

Third, this study contributes to research on team formalization by offering a more contingent account of its role in shaping team functioning. Prior literature has portrayed formalization as a double-edged structural feature. On one hand, formalization has been associated with reduced flexibility, constrained communication, and diminished knowledge exchange (Hirst et al., 2011; Amber et al., 2019; Schepers et al., 2019). On the other hand, a complementary stream of research highlights its enabling potential, suggesting that formalization can provide coordination, reduce uncertainty, and support team functioning under certain conditions (Bunderson and Boumgarden, 2010; Juillerat, 2010; Gibson et al., 2019; Fischer et al., 2019). Building on this tension, our findings suggest that the effects of formalization are best understood through a contingency perspective that considers its interplay with team-level motivational resources. Specifically, formalization does not inherently make teams more curious; rather, it appears to make curiosity more consequential for TMS development and subsequent team performance. When formalization constrains spontaneous interaction, curiosity-driven inquiry becomes a more important means for members to uncover who knows what and integrate distributed expertise. In this sense, curiosity primarily helps teams work around the limits imposed by rigid structures. At the same time, formalization may also provide clearer role boundaries and interaction channels, which can help curious members direct their inquiry more effectively once they are motivated to seek knowledge. This pattern suggests that formalization does not exert a uniform influence on team cognition, but rather conditions whether motivational resources are functionally necessary for the emergence of shared cognitive structures. Importantly, however, formalization is unlikely to be uniformly beneficial, even for curious teams. When rules are excessively rigid, strongly hierarchical, or enforced in ways that discourage questioning, team members may hesitate to challenge assumptions or probe others' expertise. Under such conditions, formalization may suppress the behavioral expression of curiosity rather than amplify its value. Thus, our argument is not that formalization always strengthens curiosity, but that moderate to high formalization can make curiosity more consequential when teams still retain sufficient latitude to engage in deliberate inquiry. This pattern resonates with De Clercq et al. (2013), who found that formalization moderated the role of organizational social capital in knowledge sharing, such that social capital mattered more when structural conditions were less conducive to knowledge exchange. Taken together, these insights extend prior work on the functional ambivalence of formalization (Gibson et al., 2019; Monteiro and Adler, 2022) by demonstrating that its effects are not fixed, but contingent on the motivational composition of the team. This perspective underscores the importance of considering the joint influence of structure and motivation in understanding team cognition and coordination.

Notably, our results revealed that the indirect effect of team curiosity on team performance via TMS was non-significant when team formalization was low. This finding warrants careful interpretation. In environments characterized by low formalization, rules and procedures are less rigid, and team members already enjoy considerable freedom to interact spontaneously, exchange knowledge informally, and engage in exploratory conversations. Under such conditions, the structural context itself facilitates the organic emergence of shared cognitive structures. As Juillerat (2010) noted, formalization is most valuable when it provides structure that is otherwise lacking; conversely, when the environment already affords flexibility and open communication, additional motivational resources such as curiosity may be less critical in driving TMS development. In other words, in low-formalization settings, team members can readily discover “who knows what” through naturally occurring interactions, without needing the additional impetus that curiosity provides. This interpretation aligns with the cognitive resource perspective advanced by Fischer et al. (2019), who found that formalization facilitated prosocial behaviors primarily in high-uncertainty contexts where cognitive resources are taxed, a logic that parallels our finding that curiosity matters most when structural constraints limit the availability of alternative pathways for knowledge exchange. Conversely, in highly formalized teams, spontaneous communication is constrained by rigid protocols and standardized procedures (Hirst et al., 2011; Mao et al., 2009), making curiosity-driven inquiry the primary vehicle through which members can overcome structural barriers and build the shared expertise maps that underpin TMS. Thus, the non-significant indirect effect under low formalization is not a null result but rather a theoretically meaningful boundary condition: it reveals that the performance benefits of team curiosity via TMS are contingent on the degree to which structural constraints create a need for curiosity-driven knowledge seeking.

Fourth, our study offered broader theoretical value by illustrating a cross-level mechanism through which individual psychological tendencies influence collective cognitive functioning and team performance. By showing how team curiosity—aggregated from individual traits—affects team performance via TMS, we helped bridge micro-level motivational psychology with macro-level team effectiveness. This multilevel perspective responds to longstanding calls in the field of organizational behavior for a stronger understanding of how individual attributes scale up to shape teams' emergent states, as well as how emergent states (e.g., TMS) serve as linking mechanisms across different levels of analysis (Ployhart and Hendricks, 2019).

### 5.2 Practical implications

Our findings have several important implications for team management and organizational design. First, our findings suggest that fostering curiosity at the team level may be beneficial for the development of TMS and, in turn, for team performance. Managers may consider not only recruit individuals with a natural propensity for curiosity but also actively cultivate a team climate that encourages inquiry, open-mindedness, and exploration. Team-level curiosity can be promoted through leadership behaviors that model curiosity (e.g., asking questions, tolerating uncertainty) as well as developmental practices like cross-training, feedback-seeking routines, and task rotations that stimulate intellectual engagement (Kashdan et al., 2023; Lievens et al., 2022).

Second, the findings highlight the importance of a TMS as a central mechanism through which team curiosity may be translated into performance. Organizations seeking to improve team coordination and cognitive integration should invest in practices that facilitate TMS development, such as structured knowledge mapping, role-clarification exercises, and regular reflection sessions to identify and align members' expertise (Yan et al., 2021). Our results underscore that motivational inputs, like curiosity, can also facilitate TMS development in teams.

Third, and perhaps most critically, our results demonstrate that team curiosity can buffer the potential downsides of high levels of team formalization. While formalized structures are often necessary to ensure clarity and efficiency, they may inadvertently stifle informal knowledge flows and spontaneous collaboration. However, when teams exhibit high levels of curiosity, they may partially overcome these constraints. Therefore, in highly formalized environments, organizations should pay close attention to maintaining and activating team curiosity as a psychological resource. This may involve creating “exploration windows” within formal routines or dedicating time to idea generation and informal knowledge exchange.

Collectively, these insights suggest that curiosity is not merely a desirable individual trait; it may function as a valuable team-level resource that, when activated under the right conditions, can significantly enhance collective cognition and performance. Therefore, managers and organizations should consider curiosity-enhancing interventions as part of their broader strategy to build agile, learning-oriented teams.

### 5.3 Limitations and future research directions

Despite its valuable contributions, this study has certain limitations—though these limitations point to potential avenues for future research. First, the data were collected from teams within a limited number of industries and cultural contexts, which may constrain the generalizability of our findings. Although our multi-industry sample provides some degree of variance in work settings, future research could examine whether the effects of team curiosity, as well as its interactions with formalization, vary by organizational environment (e.g., environments with highly dynamic, creative, or hierarchical structures). Cross-cultural studies would also help determine whether the meaning and impact of curiosity or formality-related norms are consistent across diverse national or cultural contexts.

Second, although our study used multi-source and time-lagged survey data to mitigate common method bias, the design remains correlational. As such, we cannot make definitive causal claims regarding the relationships among team curiosity, TMS, and team performance. Future research could employ experimental or longitudinal designs to examine the developmental trajectory of TMS in relation to fluctuations in team curiosity over time or to assess the causal impact of curiosity-enhancing interventions.

Third, our data were collected from organizations in China, which may shape how team curiosity and formalization operate. Cultural norms such as higher power distance, stronger respect for hierarchy, and greater adherence to formal rules may influence both the expression of curiosity and the functioning of formalized structures (Hofstede et al., 2010). In such contexts, curiosity-driven behaviors may be more salient and consequential for facilitating knowledge exchange, while formalization may more strongly constrain spontaneous interaction. As a result, the interaction between team curiosity and formalization observed in this study may be particularly pronounced in high-formality, high-hierarchy settings. In contrast, in less formal or more egalitarian contexts (e.g., many Western work environments), where open communication is more normative, the compensatory role of curiosity may be weaker or operate differently. Future research could examine the cross-cultural generalizability of these findings.

Finally, while we identified team curiosity as a novel antecedent of TMS, we did not explore other potentially interacting motivational or relational inputs. Thus, future research could examine whether psychological safety, affective tone, or leadership behaviors (e.g., leader openness or humility) further moderate or mediate the link between curiosity and TMS. Integrating such variables would offer a more holistic view of how a team's curiosity interacts with its climate and relational conditions to shape its processes and states.

## 6 Conclusion

This study introduced the concept of team curiosity as a novel motivational input that shapes how teams construct a shared cognitive infrastructure and, in turn, influences their performance outcomes. By identifying TMS as a central mechanism and team formalization as a key boundary condition, we offer a more nuanced understanding of when and how curiosity is associate with collective success. In doing so, we bridge micro-level motivational tendencies with teams' macro-level emergent states and outcomes. More broadly, our research highlights the strategic value of fostering curiosity in teams—not only as an individual virtue but as a collective resource capable of offsetting structural constraints and potentially enabling adaptive performance in complex organizational contexts.

## Data Availability

The raw data supporting the conclusions of this article will be made available by the authors, without undue reservation.
